# Evaluation of the dermatologic life quality among cleanroom workers in a secondary battery factory

**DOI:** 10.1186/s40557-016-0125-0

**Published:** 2016-09-02

**Authors:** Jae Jung Cheon, Jun Young Uhm, Gu Hyeok Kang, Eun Gye Kang, Soo Young Kim, Seong Sil Chang

**Affiliations:** Department of Occupational & Environmental Medicine, Eulji University Hospital, 95 Dunsanseo-ro, Seo-gu, Daejeon, 35233 Republic of Korea

**Keywords:** Ultra low humidity, Cleanroom, Battery factory, Skindex-29, QOL(Quality of life)

## Abstract

**Background:**

Cleanroom air is extremely dry, as it is maintained within 1 % of relative humidity. Few studies have assessed the dermatologic life quality of workers in ultralow-humidity environments. Therefore, we aimed to evaluate the dermatologic life quality of cleanroom workers using the Skindex-29, compared to those of non-cleanroom workers.

**Methods:**

Study participants were 501 cleanroom workers and 157 non-cleanroom workers from a secondary battery factory, who underwent an employee health examination at a single university hospital from September 2014 to September 2015. Results of the self-administered Skindex-29, and McMonnies questionnaire were analyzed. Other information and disease history were also collected during physician's medical examination. Descriptive and multivariate logistic regression analysis were performed.

**Results:**

The Skindex-29 score was significantly higher in cleanroom workers than in non-cleanroom workers for all domains, Symptom (16.0 ± 15.9 vs. 6.3 ± 10.2, *p* < 0.001), Emotion (11.3 ± 17.4 vs. 2.5 ± 7.4, *p* < 0.001), Function (5.2 ± 11.1 vs. 1.6 ± 4.0, *p* < 0.001), and Overall (10.8 ± 13.4 vs. 3.5 ± 6.2, *p* < 0.001). The Skindex-29 score of cleanroom workers was similar to that of patients with skin diseases such as psoriasis, other dermatitis, corns, alopecia etc. Among the cleanroom workers, 37 workers had one or more skin diseases.

Among the risk factors, ‘working at cleanroom’, ‘possessing skin disease’ and ‘McMonnies score’ had significant strong correlations with Skindex-29 score, meanwhile age, sex, smoking, drinking and exercise had weak correlations with it.

‘Working at cleanroom’ and ‘possessing skin disease’ had highest odds ratios with overall 14.0 (C.I.: 5.9–33.1) and 13.4 (C.I.: 4.5–29.2), and the lowest odds ratios with function domain 3.5(C.I.: 1.7–7.1) and 4.5(C.I.: 2.1–9.5), respectively. The McMonnies score had the highest odds ratio with overall, 6.9(C.I.: 4.5–10.8) and lowest odd ratio with emotion domain 4.2 (C.I.: 2.7–6.4).

**Conclusions:**

Dermatologic life quality among cleanroom workers in the secondary battery factory is shown to be lower than that among non-cleanroom workers in this study. The study suggests that the Skindex-29 may provide helpful information on the dermatologic life quality of cleanroom workers. Therefore, regarding evaluation of dermatologic life quality using Skindex-29, preventive care is necessary for cleanroom workers in ultralow humidity environment.

## Background

In Korea, the cleanroom industry was started by conglomerates’ investment high technology industries such as semiconductor companies since 1980. It is estimated that the market size of the cleanroom industry has increased from 200 billion in the early 2000-s to 1 trillion Korean won at present [1]. Additionally, the number of industries that operate cleanrooms for manufacturing fine products such as electronics, precise machinery, pharmaceuticals, food companies, etc. has been rapidly increased.

Cleanroom is a special work environment, of which the air is maintained artificially clear by controlling the number of particles and the temperature, moisture, pressure as well. The American Society of Heating, Refrigerating and Air-conditioning Engineers (ASHRAE) reported that the optimal temperature and relative humidity for quality living are 17–28 °C and 40–70 %, respectively [2].

Maintaining extremely low humidity in a cleanroom is also essential for manufacturing lithium ion batteries. The range of humidity in a cleanroom is maintained at less than 1 %, which is main risk factor of adverse health effects on the eyes, skin, and respiratory mucosa. Among the manufacturing processes mixing, coating, roll pressing, slitting, punching, vacuum drying, assembling, folding, lot number marking, formation and grading, and shipping - vacuum drying and assembling require an ultra-low concentration of humidity and the elimination of dust.

There have been several Korean studies on the adverse health effects on cleanroom workers. Particles, chemicals, shift-work were considered main hazardous factors [3], and dry eye syndrome was a main health problems [4]. Working in a space with a low humidity can cause dryness of the eyes and skin and sick building syndrome [5], which is characterized by discomfort, fatigue, headache, or myalgia. In a study on flight attendants who worked on airplanes where the relative humidity 14–19 %, complains of dry eye, dry skin and nasal mucosa were common [6]. Another study compared flight attendants who worked on the airplanes where the relative humidity 5–25 % to common office workers in a general office. The odds ratios of facial and hand skin discomfort were 2.03 and 3.68, respectively higher among flight attendants than those of office workers [7]. Working in a low humidity environment is a risk factor of sick building syndrome [5], and main complaints are eyes or skin dryness and symptoms such as fatigue, headache, or myalgia. General and fatal disorders of the urinary and cardiovascular system are aggravated by the dry condition. If the dry condition persists, the body becomes dehydrated, the osmotic pressure of plasma and urine increases, which can cause deep vein thrombosis and pulmonary embolism [8]. In some bio-molecular studies, filagrin, a structure of the stratum corneum, is destroyed when exposed to a condition in which the relative humidity is less than 10 % [9]. Epidermal DNA synthesis increases and epidermal hyperplasia makes the stratum corneum thicker when the relative humidity is maintained at less than 10 % with dry air [10]. Chemical changes in the skin wall precede physical changes of the skin, and a dry condition causes hyperplasia and degranulation of dermal mast cells, which cause an inflammatory reaction in environments with a relative humidity less than 10 % [11]. As aforementioned, a dry condition cause physical and chemical defects on the skin wall and affects skin's function, which further causes dermatologic symptoms such as dryness, itching, and a burning sensation [12]. The percentage of dermatologic symptom of cleanroom workers in a high-technology device developing laboratory, where the relative humidity maintained at less than 2.5 % was about 2.6 times higher (42 % vs. 16 %) than that of workers in a general environment(relative humidity 40–70 %), and the prevalence of atopic dermatitis was also significantly higher (33 % vs. 6 %) [13]. Although many symptoms of discomfort are prevalent and they can progress to chronic skin diseases, no systemic screening or monitoring has been done for evaluating the health risk of the deteriorating dermatologic life quality of cleanroom workers.

The Skindex-29, designed by Chren et al. in 1996, can be applied as an effective screening/monitoring tool for cleanroom workers with skin symptoms or skin diseases since it has been shown fairly reliable, reproducible, and valid data among dermatologic diseased population [14]. By minimizing the floor effect, the Skindex-29 has been known more sensitive assessment tool than other quality of life assessment tools such as the Dermatology Life Quality Index (DLQI), or Psoriasis Disability Index (PDI), health related quality of life (HRQOL), and Medical Outcome Study Short Form 36 (SF-36) [15].

Most skin symptoms are not fatal, so they are often neglected. However, significant dermatologic symptoms should be properly assessed and prevented before they progress to a chronic skin disease. Since an ultralow-humidity environment affects workers’ dermatologic life quality and causes skin discomfort before chronic diseases develop, we need effective screening/monitoring tools to assess the severity of skin symptoms and prevent them from progressing to a chronic skin disease or causing a decreased life quality. Therefore, using Skindex-29, we evaluated how serious the dermatologic symptoms and life quality among cleanroom workers who work in a very dry environment, and compared those to workers’ in general environment.

## Methods

### Study participants

Five hundred one workers who worked in a cleanroom where relative humidity is controlled within 1 % in a secondary battery producing factory from September 2014 to September 2015 were enrolled in this study. To minimize such confounding factors, 157 non-cleanroom workers from the same factory, were enrolled for comparison.

Sjogren syndrome is an important confounding autoimmune disease that also causes dry eye and skin dryness. Sjogren syndrome affects no more than 3.9–5.9 cases per million people, and the disease affects those in their 40s to-50s [16]. Sjogren syndrome worsen patient’s life quality as it’s symptoms include eye and skin dryness, fatigue, arthralgia, and myalgia [17]. Sjogren syndrome can be diagnosed in patients with subjective symptoms such as eye, oral and skin dryness if more than two of the three objective criteria are fulfilled [18]. 1) Abnormal hematologic findings such as positive serum anti-SSA, anti-SSB, rheumatoid factor and ANA results. 2) Ocular staining score ≥ 3. 3) Pathologic findings such as the presence of focal lymphocytic sialadenitis. On history taking during medical examination, no workers in this study have been diagnosed as Sjogren syndrome. Therefore, no participants were excluded on the behalf of Sjogren syndrome.

### Measures

All subjects were asked to complete a questionnaire regarding general data such as age, sex, work duration, position, and dermatologic issues such as skin discomfort and history of dermatologic diseases. In addition, an occupational environmental medicine physician performed a medical examination by interview and inspection. The Skindex-29, Korean was used to evaluate the severity of dermatologic life quality, with three domains --7 questions about symptom, 10 about emotion, 12 about function-- and an overall score, the sum of all three domains. Each question was scored from 0 to 4 as follows: 0. never, 1. rarely, 2. sometimes, 3. often, and 4. always. The total score ranged from 0(never for all question) to 100(always for all questions), and higher score indicates lower life quality [19].

### Statistical analysis

SPSS ver. 18 window version was used to perform statistical analysis. The Skindex-29 score between the cleanroom workers and non-cleanroom workers was compared by using the student *t*-test. The chi-square was used to perform cross tabulation between the elder and younger age group of the cleanroom workers. The Pearson correlation used to analyze the association among workers’ profiles (age, sex, skin disease, smoking status, alcohol consumption, exercise, McMonnies score and working place, work duration, working hour, position, working form--blue/white) and the total score or each domain score of Skindex-29. Multivariate logistic regression analysis was used to calculate odds ratio of the high Skindex-29 score group among the risk factors. Two-sided *p*-value less than 0.05 was considered statistically significant.

## Results

General characteristics of the cleanroom workers and non-cleanroom workers are presented in Table 1.

**Table 1:** General characteristics of cleanroom workers and non-cleanroom workers, and other surveyed characteristics of cleanroom workers

Characteristics	Cleanroom	Non-cleanroom
Number	501	157
Age^**^ (mean ± SD)	31.2 ± 6.6	35.1 ± 9.0
Gender^*^(n, %)		
Male	499 (99.7)	150 (95.6)
Female	2 (0.3)	7 (4.4)
^a^Skin disease (n, %)	37 (7.4)	9 (5.7)
Smoking (%)	43.1	35.8
^b^Alc. drinking^*^(%)	27.8	17.5
^c^Non-exercise (%)	24.8	21.7
McMonnie questionnaire (mean ± SD)	6.5 ± 4.3	
^d^Working duration (mean ± SD)	1752.0 ± 1442.7	
^e^Working hours(mean ± SD)	53.1 ± 19.0	
^f^Position(plain/manager, %)	90.0/ 10.0	
^g^Working form(Blue/White)	98.0/ 2.0	

^*^ *p* < 0.05, ^**^ *p* < 0.001

^a^Dermatologic diseases (cleanroom/non cleanroom) pruritus (8 cleanroom workers /1 control group), acne (4/1), seborrheic dermatitis (3/1), atopic dermatitis (1/1), tinea pedis (1/1), allergic contact dermatitis (1/1), xeroderma (3/0), dermographism (1/0), ecthyma (1/0), hives (1/0), corn (1/0), tinea cruris (0/1), psoriasis (0/1), and keloid (0/1)

^b^High risk groups of people drinking more than 15 glasses of alcohol on a week

^c^Non Exercise group of people exercising less than 600METs on week

^d^Working period(days) in the cleanroom

^e^Mean working hours per week in the cleanroom

^f^Plain: Regular workers who were supervised by manager

Manager: supervisor of regular workers

^g^White workers are office workers

Blue worekrs are workers actually involving in manufacturing products

The proportions of male workers in the cleanroom workers and non-cleanroom workers were 99.7 % and 95.6 %, respectively (*p* < 0.05). The mean age of clean and non-cleanroom workers were 31.2 ± 6.6 and 35.1 ± 9.0 years (*p* < 0.001). The proportions of current smokers were 43.1 and 35.8 % for the cleanroom workers and non-cleanroom workers, respectively (*p* = 0.144). The standard cut off criteria of high risk heavy drinker group from Korean National Health and Nutrition Examination Survey (KNHANES) is defined as ‘more than twice in a week and more than 7 glasses for men and 5 glasses for women at a time’. The proportions of high risk heavy drinkers in the cleanroom workers and non-cleanroom workers were 27.8 and 17.5 %, respectively (*p* < 0.05). The proportions of cleanroom workers and non-cleanroom workers in the a non-exercise group, defined as a group of people who exercised less than 600MET-minutes [20] were 24.8 and 21.7 %, respectively (*p* =0.458).

The prevalence rates of dermatologic diseases in the cleanroom workers and non-cleanroom workers were 7.4 and 5.7 % respectively (*p* > 0.05). Dermatologic diseases (among the cleanroom workers/non-cleanroom workers) included pruritus (8/1), acne (4/1), seborrheic dermatitis (3/1), atopic dermatitis (1/1), tinea pedis (1/1), allergic contact dermatitis (1/1), xeroderma (3/0), dermographism (1/0), ecthyma (1/0), hives (1/0), corns (1/0), tinea cruris (0/1), psoriasis (0/1), and keloids (0/1).

Regarding occupational features of the cleanroom workers, the mean work duration was 1752 ± 1442.7 days and the mean working hours per week was 53.1 ± 19.0 h. The proportions of plain workers and managers were 90.0 and 10.0 %, respectively. The proportions of blue-collar workers and white-collar workers were 98.0 and 2.0 % respectively.

McMonnies questionnaire is a survey related to clinical factors of dry eye, which is used to diagnose dry eye and to evaluate the severity. The total score of this survey is 24. If the score is same as or higher than 10, it is abnormal, it the score is lower than 10, in contrast, it is normal [21]. In this study, the McMonnies score was 6.5 ± 4.3, which is lower estimated than the score of the previous study on health college students [22].

Comparison of the Skindex-29 score between the cleanroom workers and non-cleanroom workers is presented in Table 2. The Skindex-29 scores were significantly higher in cleanroom workers than in non-cleanroom workers for all three domains and overall score, symptom (16.0 ± 15.9 vs 6.3 ± 10.2, *p* < 0.001), emotion (11.3 ± 17.4 vs 2.5 ± 7.4, *p* < 0.001), function (5.2 ± 11.1 vs 1.6 ± 4.0, *p* < 0.001), and overall (10.8 ± 13.4 vs 3.5 ± 6.2, *p* <0.001).

**Table 2:** Skindex-29 Score of both groups

^a^Scale	Cleanroom (*n* = 501)	Non-cleanroom (*n* = 157)	*p*-value
Symptom	16.0 ± 15.9	6.3 ± 10.2	<0.001
Emotion	11.3 ± 17.4	2.5 ± 7.4	<0.001
Function	5.2 ± 11.1	1.6 ± 4.0	<0.001
Overall	10.8 ± 13.4	3.5 ± 6.2	<0.001

^a^Skindex-29 have three domains: symptom, emotion, function, and overall score as for the sum of all domains

Regarding Association between skin diseases and age groups among the cleanroom workers, age group was divided into two groups, junior (20–39 years old) and senior (40 years old or elder). All 37 workers who have been suffered from skin diseases, were under 40 years old among 501 cleanroom workers (*p* < 0.05, Fisher exact test).

Correlation between the worker's profiles and Skindex-29 scores among the cleanroom workers is presented in Table 3. Regarding intensity of correlation coefficient, 0.7 ≤ r were considered as a strong correlation, 0.3 ≤ r < 0.7, a moderate correlation, and 0.1 ≤ r < 0.3, a weak correlation. Demographic factors such as age, sex, and life style such as smoking, drinking and exercise were not significantly correlated with the Skindex-29. Moderate positive correlations were found between the prevalence of ongoing dermatologic diseases and the Skindex-29score for the emotion domain and overall score, and weak positive correlations between two variables in Symptom and Function domains. Moderate positive correlations were observed with the McMonnies score for all domains of Skindex-29. Working in a cleanroom had weak positive correlations with all three domains and overall score. Other occupational factors such as the working duration, mean working hours per week, position, and shift-work were not significantly correlated with any domain of the Skindex-29.

**Table 3:** Correlation coefficients between worker’s profile and Skindex-29 score

	Symptom	Emotion	Function	Overall
Age	−.047	−.109^*^	−.030	−.075
Gender	−.016	.025	.036	.027
Skin disease	.292^*^	.315^*^	.264^**^	.326^**^
Smoking	−.061	−.086^*^	−.055	−.077
Alc. Drinking	.059	−.109^*^	−.100^*^	−.098^*^
Non -exercise	−.123^**^	−.052	−.050	−.086^*^
McMonnie questionnaire	.471^**^	.410^**^	.322^**^	.453^**^
^a^Cleanroom	.268^**^	.232^**^	.152^**^	.250^**^
Working period	.043	.003	.080	.040^*^
Working hours	.009	.025	−.057	−.001
Position (plain/manager)	.015	−.062	−.039	−.032
Working form (Blue/White)	−.060	−.053	−.002	−.047

*p < 0.05, **p < 0.001

^a^Whether or not a worker works in the cleanroom

Odds ratios of demographic factors and worker’s profiles to the Skindex-29 score are presented in Table 4. The odds ratios were 5.56(C.I.: 3.23–9.56), 8.54(C.I.: 3.80–19.19), 3.52(C.I.: 1.73–7.15), and 14.04(C.I.: 5.95–33.12) for symptom, emotion, function, and overall, respectively, which showed cleanroom workers had higher risk than non-cleanroom workers. Workers with a current skin disease had 9.750(C.I.: 3.59–26.48), 5.15(C.I.: 2.34–11.31), 4.52(C.I.: 2.14–9.53), and 13.45(C.I.: 4.59–29.29) times higher odds ratios for symptom, emotion, function, overall, respectively than those of workers with no current skin disease.

**Table 4:** Odds ratio (95 % CI) of demographic factors and worker’s profiles to skindex-29 score

	SkindexSx.	SkindexEm.	SkindexFx.	Skindex Overall
Cleanroom	5.56 (3.23–9.56)	8.54 (3.80–19.19)	3.52 (1.73–7.15)	14.04 (5.95–33.12)
Skin Disease	9.75 (3.59–26.48)	5.15 (2.34–11.31)	4.52 (2.14–9.53)	13.45 (4.59–29.29)
^a^McMonnie grade	5.37 (3.58–8.05)	4.20 (2.74–6.44)	5.48 (3.36–8.94)	6.98 (4.50–10.83)

All the test *p value* were <0.001

^a^McMonnie grade is subdivided into high risk group and low risk group using average score

When the McMonnies score was subdivided into the high-risk group and low-risk group using an average of the cut off value. Workers with higher McMonnies score had 5.37(C.I.: 3.58–8.05), 4.20(C.I.: 2.74–6.44), 5.48(C.I.: 3.36–8.94), 6.98(C.I.: 4.50–10.83) times higher Skindex-29 score compared to those of workers with lower McMonnies score. Other worker’s profiles such as work duration, weekly work hours, and total working hours during the last week were not significantly associated with an increasing odds ratio in terms of Skindex-29 scores.

## Discussion

In modern days, the concept of health is focused not just on the condition of no diagnosed diseases, but on the quality of life. The World Health Organization defines quality of life as people's perception of their position in life in the context of the culture and value systems, in which they live and in relation to their goals, expectations, standards and concerns [23]. There are two types of tools for assessing life quality; health related quality of life (HRQOL) and non-health related quality of life(NHRQOL). The HRQOL evaluates the factors that affect an individual's health such as diseases, which affects life quality. The NHRQOL assess the complex reaction of personal, internal and environmental factors such as health, salary, climates and living environment that affect the life quality.

Personal factors such as depression, the sense of accomplishment and a positive mind set affect the job stress of workers, whereas the working conditions such as the intensity, salary, welfare system, company atmosphere, and climate affect worker's subjective symptoms [24]. Job stress can causes psychiatric disorders such as depressive disorder or negative emotion, and cardiovascular disorder such as coronary heart disease or musculoskeletal diseases such as epicondylitis, in addition, job stress affects the immune system by regulating T lymphocytes and secreting interleukin(IL)-2,4 [25]. A previous study showed that workers with a low job satisfaction visit clinics and hospitals more than twice as many times as workers without low job satisfaction [26]. Therefore, job stress caused by multiple factors that causes psychiatric and physical health problems, affect workers’ life quality. Thus, factors other than working in a cleanroom should have been controlled in this study. Non-cleanroom workers in the same factory were designated as the participants of this study in order to control confounding factors such as salary, welfare, company culture and atmosphere.

In this study, the Skindex-29 score was significantly higher in the cleanroom workers than in the non-cleanroom workers in all domains of Skindex-29. Moreover, working in a cleanroom was a risk factor decreasing dermatologic life quality. The Skindex-29 scores of cleanroom workers in this study were similar or even higher than those in their 20s with psoriasis, other dermatitis, viral warts, corns and androgenic alopecia or patients with onychomycosis, pityriasis versicolor, alopecia areata, pompholyx, and naildystrophy [27]. These findings suggest that the quality of life of workers in a cleanroom might be similar to or worse than that of a similar age group of men with dermatologic diseases.

Regarding the demographic factors, participants in this study had healthier life styles (i.e., fewer smoker, drinker, and non-exerciser) than those of similar age groups in the general Korean population. The McMonnies score in our study was also lower than that reported in a previous study. Despite such health worker effects in this study, the symptom scores were much higher than among workers than the general population [28].

Remarkably, 37 cleanroom participants with existing skin diseases were all younger than 40 years. Young age was a risk factor of skin diseases in cleanroom workers in a Taiwan study [29]. The senior group in this study had less than 1.0 odds ratios in overall Skindex-29 score compared to the younger than 40 years old, which indicated that the risk of skin symptoms may be decreased by aging or healthier workers be selected survival for longer working at the cleanroom [30].

McMonnies questionnaire is a survey tool relevant to self-perceived symptoms such as eyeball dryness and pain. Thyroid disease and arthritis, drugs inducing the dry eye syndrome such as antihistamine agents, antihypertensive drugs can induce dry eyes’ symptoms. McMonnies score had a moderate positive correlation with all domains of the Skindex-29. Workers with higher McMonnies score had 4.2–6.9 times higher scores in all three domains and overall score of Skindex-29 compared to those with lower score. In our study, workers in dry environments have more subjective skin and eyeball discomforts according to the Skindex-29 and McMonnies questionnaire than non-cleanroom workers had. Even though those are screening methods for subjective symptoms, these results also suggest that dry environments can affect the skin and eyeballs symptoms/diseases, and as a consequence, one's life quality can be affected.

Skin disease had a moderate correlation with emotion domain and overall score of Skindex-29. Furthermore, workers with a current skin disease had 4.5–13.4 times higher risk of severe symptoms than that of workers without skin diseases in all three domains and overall score of Skindex-29. Skin disease is closely associated with the dermatologic life quality [31]. Therefore, patients with skin diseases working in a cleanroom should be cared and treated properly.

A study on patients with atopic dermatitis reported that life quality was not associated with age and sex [32]. Whereas another study on patients with contact dermatitis reported that age but not sex was associated with the quality of life [33]. The prevalence rate of skin diseases in male adult workers at an orchard was 6.5 %, which is similar to those reported in recent studies, but it was much lower than the prevalence of skin diseases in adults in Seoul, Korea [34, 35]. The prevalence rate of adult atopic dermatitis was 3–4 % in another study [36], which was not different from that for atopic dermatitis in this study. Although the prevalence of dermatologic diseases has not been consistent on the basis of the investment method and study populations, considering the healthy-workers’ effect on this young study population, the prevalence of skin disease or atopic dermatitis of the cleanroom workers should have been much lower than that of a general reference population. If the prevalence rate was similar or higher, it may have been affected artificially by their work environment.

This study has some limitations. First, most subjects were young men in their 20s to-30s. Women and seniors may be more vulnerable to dermatologic symptoms and diseases under such an ultralow humidity work condition. Second the diagnostic tool for assessing the dermatologic life quality was a subjectively administered questionnaire. Compared to a specialist's perception of symptoms, the degree of an individual's perception of dermatologic symptoms can be more exaggerated [37]. Dry environments destroy the skin barrier physically and chemically, and weaken the overall function of the skin barrier. They also accelerate the secretion of hormones such as cortisol and pro-inflammatory cytokines such as IL-2, and increase the number of mast cells, which can result in allergic reactions such as pruritus, rash or burning sensation [38]. Thus, it is likely that workers in poor working conditions overstate their skin symptoms. Therefore, further studies might be helpful if it could include objective skin indices such as the humidity of the skin, degree of wrinkles and elasticity as well as dermatologic diagnosis by professionals.

Despite these limitations, our findings are very significant and original since there have been few studies on dermatologic problems and quality of life in those who work in extremely dry environments with 1 % or lower relative humidity. Since the number of indoor humidity controllers such as air-conditioners is increasing and the amount of work in a cleanroom is expected to be increasing as industrials have been developed. The health environmental significance of this study will be concerned to more people from the workers to the health practitioners and company owners. Active and integrative health management for preventing diseases caused by dry environments is essential. And exposure to dry environments itself should be minimized by segmenting the work process, reducing processes in a cleanroom by replacing robots system to perform the work in extremely dry conditions, providing enough interval times for workers to take breaks, or encouraging shift-work. But many hazardous working environments still affect the dermatologic life quality of workers. Thus, a future study is needed to verify the effectiveness of Skindex-29 for evaluating the dermatologic life quality among general population and different workers exposed to various organic solvents, chemicals, physical factors and mental stress.

## Conclusions

An artificially dry condition may cause dryness and discomfort in the eyes, skin and mucosa. Itching and a stinging sensation on the skin are main symptoms and such symptoms can be risk factors of a worsening life quality. In this study, we investigated the life quality of workers in the cleanroom with ultra-low humidity being controlled within 1 % of relative humidity through Skindex-29. The life quality of Cleanroom workers was evaluated lower than those of non-cleanroom workers in all three domains and overall score. Pre-existing skin disease or accompanying dry eye syndrome showed good correlations with and higher odds ratio to dermatologic symptoms evaluated by all domains of Skindex-29. The Skindex-29 and McMonnies questionnaire were helpful for evaluating the dermatologic/ophthalmologic life quality of cleanroom workers and these tools can be used to further monitor effects of health management plans, such as supplying protective clothes and skin moisturizer, managing self-sanitation, and following proper intervention of break-time, regular dermatologic/ophthalmologic check-up, and etc. We suggest that the Skindex-29 might be an effective tool for evaluating dermatologic problems among other occupational settings that cause skin problems and affect the life quality of workers, and dermatologic proper health management is necessary for cleanroom workers in ultralow humidity environment to improve their life quality.
